# Transient ECM protease activity promotes synaptic plasticity

**DOI:** 10.1038/srep27757

**Published:** 2016-06-10

**Authors:** Marta Magnowska, Tomasz Gorkiewicz, Anna Suska, Marcin Wawrzyniak, Izabela Rutkowska-Wlodarczyk, Leszek Kaczmarek, Jakub Wlodarczyk

**Affiliations:** 1 Department of Molecular and Cellular Neurobiology, Nencki Institute, Pasteura 3, Warsaw, 02-093, Poland; 2 Department of Neurophysiology, Nencki Institute, Pasteura 3, Warsaw, 02-093, Poland; 3 Department of Physics, Warsaw University of Life Sciences, Nowoursynowska 159, Warsaw, 02-776, Poland

## Abstract

Activity-dependent proteolysis at a synapse has been recognized as a pivotal factor in controlling dynamic changes in dendritic spine shape and function; however, excessive proteolytic activity is detrimental to the cells. The exact mechanism of control of these seemingly contradictory outcomes of protease activity remains unknown. Here, we reveal that dendritic spine maturation is strictly controlled by the proteolytic activity, and its inhibition by the endogenous inhibitor (Tissue inhibitor of matrix metalloproteinases-1 – TIMP-1). Excessive proteolytic activity impairs long-term potentiation of the synaptic efficacy (LTP), and this impairment could be rescued by inhibition of protease activity. Moreover LTP is altered persistently when the ability of TIMP-1 to inhibit protease activity is abrogated, further demonstrating the role of such inhibition in the promotion of synaptic plasticity under well-defined conditions. We also show that dendritic spine maturation involves an intermediate formation of elongated spines, followed by their conversion into mushroom shape. The formation of mushroom-shaped spines is accompanied by increase in AMPA/NMDA ratio of glutamate receptors. Altogether, our results identify inhibition of protease activity as a critical regulatory mechanism for dendritic spines maturation.

Strictly controlled proteolysis plays a fundamental role in a variety of cellular and physiological phenomena. However, excessive proteolytic activity is detrimental to the cells and tissues. There are numbers of means to prevent excessive proteolysis in the cell^1^. A good example might be provided by extracellular matrix metalloproteinases (MMPs). They are tightly regulated at the levels of gene transcription, mRNA stability, local delivery and translation and then the proteins are produced in a latent form, released from the cells to unleash their enzymatic activities only after the propeptide is cleaved off 2. When proteolytically active, they are subjected to a variety of local protein inhibitors, such as TIMPs – tissue inhibitors of MMPs that nullify their activity. Intuitively, it has been widely assumed that main role of this inhibition is to prevent excessive, cell-detrimental proteolytic activity. In the present study we reveal a novel function of MMP inhibition in promoting synaptic plasticity.

Synaptic plasticity is the ability of the adult brain to modify synaptic strength and remodel neuronal circuits^3^,^4^. Dendritic spines are small, neuronal protrusions that harbor excitatory synapses. They have been recognized as critical loci of change that underlie synaptic plasticity. Spines undergo morphological changes in response to stimuli that modulate neuronal activity. Such remodeling supports the formation and long-term storage of information in the brain^3^,^4^,^5^,^6^, whereas alterations in spine remodeling frequently accompany neurodegenerative and neuropsychiatric diseases^7^,^8^.

MMP-9, a major metalloproteinase expressed in the brain, was shown to have an important role for physiological synaptic plasticity, i.e., the ability of the adult brain to modify synaptic strength and remodel neuronal circuits underlying learning and memory, by controlling the shape and efficacy of excitatory synapses in the brain^9^,^10^,^11^,^12^,^13^,^14^. MMP-9 is activated and indispensable for synaptic potentiation (including long-term potentiation of synaptic efficacy, LTP)^9^,^11^,^15^,^16^ and thus belongs to pivotal modulators of dendritic spines shape (for more extensive review please refer^17^). At the excitatory synapses MMP-9 may cleave several substrates whose function might be associated with changes in synaptic plasticity, such as β-dystroglycan, ICAM-5, neuroligin-1, nectin-3^16^,^18^,^19^,^20^,^21^,^22^.

Previously, differential if not apparently contradictory, effects of MMP-9 on dendritic spines have been reported. Whereas Michaluk *et al*.^23^, Tian *et al*.^20^ and Bilousova *et al*.^24^ have shown that excessive MMP-9 activity produces dendritic spine elongation, Wang *et al*.^11^ and Szepesi *et al*.^12^ reported enhanced spine maturation into mushroom-shaped spines under conditions of enhanced MMP-9 activity.

To resolve this apparent discrepancy, we hypothesized that regulation of transient proteolysis and by timing of its activator and inhibitors might be one of the key mechanisms that control synaptic plasticity. We report herein that ***transient*** proteolysis at the synapse initiates the promotion of structural and functional plasticity, and the ***subsequent inhibition*** of proteolysis by TIMP-1 is a key cause of dendritic spine maturation and maintenance of long-term potentiation of synaptic efficacy (LTP).

## Results

### Enzymatic activity of recombinant auto-activating protease, MMP-9 initiates morphological changes in dendritic spines that are concluded by the subsequent inhibition of proteolytic activity

**Figure 1:**
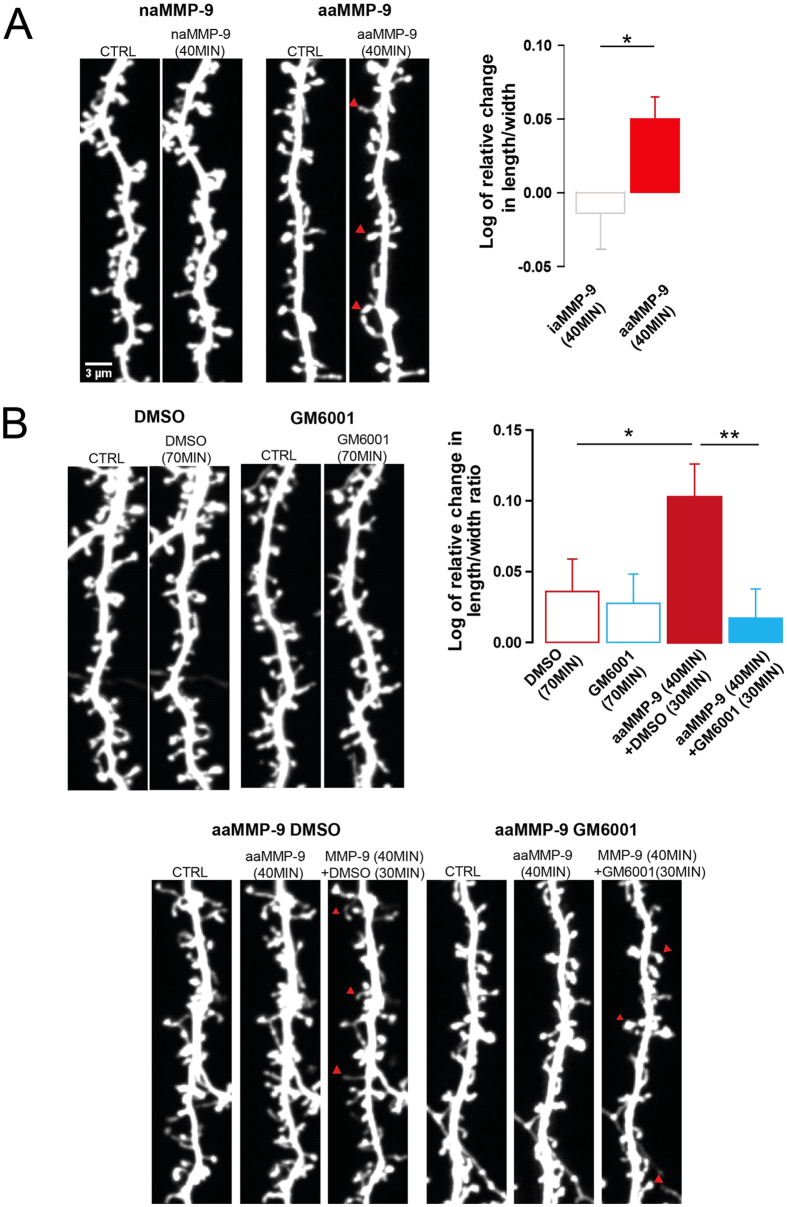
Enzymatic activity of recombinant auto-activating MMP-9 initiates morphological changes in dendritic spines that are concluded by the subsequent inhibition of proteolytic activity. (A) Representative images of live cell imaging of 21 DIV hippocampal neurons that expressed EGFP. Dendrites were imaged before (CTRL) and after treatment (40 minutes of incubation with iaMMP-9/aaMMP-9). Examples of spines that became elongated after MMP-9 activity are indicated by red arrows. Relative changes (mean ± SEM) in the length/width ratio of dendritic spines 40 min after iaMMP-9 or aaMMP-9 treatment. Incubation with iaMMP-9 did not induce prominent changes in dendritic spine shape (*n*_spine_ = 267; length/width, -0.014 ± 0.024), whereas 40 min incubation with aaMMP-9 caused elongation of the spines (*n*_spine_ = 838; length/width, 0.050 ± 0.015; *p* = 0.02). (B) Representative images of live cell imaging of 21 DIV hippocampal neurons that expressed EGFP. Dendrites were imaged before (CTRL) and after treatment (40 minutes of incubation with aaMMP-9 and then GM6001/DMSO for 30 minutes, 70 minutes with GM6001/DMSO).The inhibition of MMP activity by GM6001 blocked elongation of the spines (*n*_spine_ = 488; length/width, 0.017 ± 0.020; *p* = 0.006). The GM6001 solvent (DMSO) did not stop the elongation process induced by aaMMP-9 application (*n*_spine_ = 370; length/width, 0.103 ± 0.023). Comparing to control (incubation with DMSO itself for 70 min) the incubation with aaMMP-9+DMSO induced significant changes in dendritic spine length/width (*n*_spine_ = 266; length/width, 0.036 ± 0.021; *p* = 0.0425). The application of GM6001 for 70 minutes did not induce a significant change in dendritic spines morphology (*n*_spine_ = 339; length/width, 0.028 ± 0.019).

We previously showed that activity of exogenously applied autoactivating mutant of MMP-9, aaMMP-9 provoked the elongation of dendritic spines^23^. In the present study, we first investigated whether the elongated morphology of dendritic spines that is caused by MMP-9 activity can be affected by inhibiting the enzyme. To induce the elongation of dendritic spines, recombinant aaMMP-9 was exogenously applied to dissociated hippocampal cultures. As a control, enzymatically inactive mutant of MMP-9 (iaMMP-9) was applied. Figure 1A shows representative images of enhanced green fluorescent protein (EGFP)-transfected dendrites that are decorated with dendritic spines before and after aaMMP-9/iaMMP-9 application. The changes in spine shape were observed, including those in opposite directions that can be explained by the spontaneous intrinsic fluctuation of dendritic spine shape^25^. However, the detailed quantification of relative changes in spine shape (Fig. 1B) demonstrated that 40 min incubation of neurons with aaMMP-9 made a significant fraction of the spines longer and thinner, as compared with the negative control iaMMP-9 (as previously demonstrated^23^). Next, we investigated whether changes in the morphology of dendritic spines can be affected by subsequently applying a general inhibitor of MMPs (GM6001) 40 min after aaMMP-9 treatment (Fig. 1A,B). The inhibition of exogenously applied MMP-9 by GM6001 resulted in a marked change in spine morphology, making them shorter and wider when compared with the control experiment, in which after 40 min treatment with aaMMP-9, the inhibitor solvent was applied. The changes in dendritic spines morphology were not observed in control experiments where only GM6001 or DMSO was administrated. These results show that an overabundance of MMP-9 activity produces spine elongation, but its subsequent inhibition results in spine maturation, reflected by spine head expansion.

### Protease inhibition in transgenic rats that manifests excessive proteolytic activity (i.e. overexpress MMP-9) induces the maturation of dendritic spines

**Figure 2:**
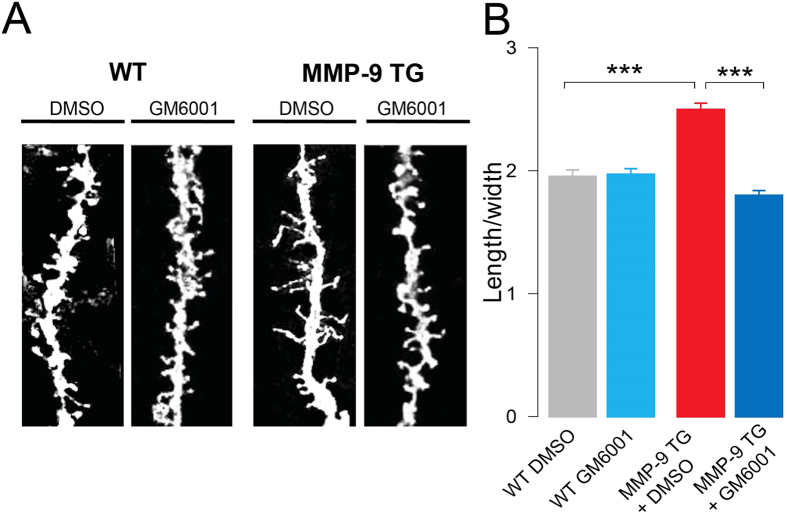
MMP-9 inhibition in transgenic rats that overexpress MMP-9 induces the maturation of dendritic spines. **(A)** Representative images of fragments of DiI-stained dendrites in the CA1 area of the hippocampus from transgenic rats that overexpressed aaMMP-9 (MMP-9 TG) and wild type (WT) animals. The dendritic spines of MMP-9 TG rats were longer and thinner compared with WT. Application of the MMP inhibitor GM6001 caused the maturation of dendritic spines in TG animals, but it did not affect the shape of dendritic spines in WT animals. **(B)** The bar plot shows a significant decrease in the length/width ratio of dendritic spines in MMP-9 TG rats after GM6001 administration (*n*_rats_ = 4; length/width, 1.72 ± 0.04; *p* < 0.001) compared with dendritic spines in DMSO-treated MMP-9 TG rats (*n*_rats_ = 3; length/width, 2.35 ± 0.056). The dendritic spines of MMP-9 TG rats were significantly longer and thinner (*n*_rats_ = 3; length/width, 2.35 ± 0.056; *p* < 0.001) compared with WT in the control state (DMSO; *n*_rats_ = 4; length/width, 1.95 ± 0.054).

To confirm the importance of inhibiting MMP-9 activity to increase spine head size (i.e., spine maturation) *in vivo*, we analyzed the shape of dendritic spines in hippocampal slices of MMP-9 transgenic rats (MMP-9 TG) that were incubated with the MMP inhibitor GM6001 (Fig. 2A). We measured the length/width ratio of DiI-stained dendritic spines (Fig. 2A). Figure 2B presents the morphometric analysis of dendritic spines in MMP-9 TG rats, which were significantly longer and thinner than in wild type animals (as previously demonstrated^23^). The application of GM6001 induced a robust decrease in the length/width ratio of dendritic spines in MMP-9 TG rats, resulting in shorter and wider spines, acquiring mushroom shape. Strikingly, MMP inhibition with GM6001 did not induce morphological changes in dendritic spines in wild type animals. These results showed that endogenously produced excessive MMP-9 activity caused spine elongation, but its inhibition allowed for spine maturation.

### Protease inhibition recruits AMPARs to synapses

**Figure 3:**
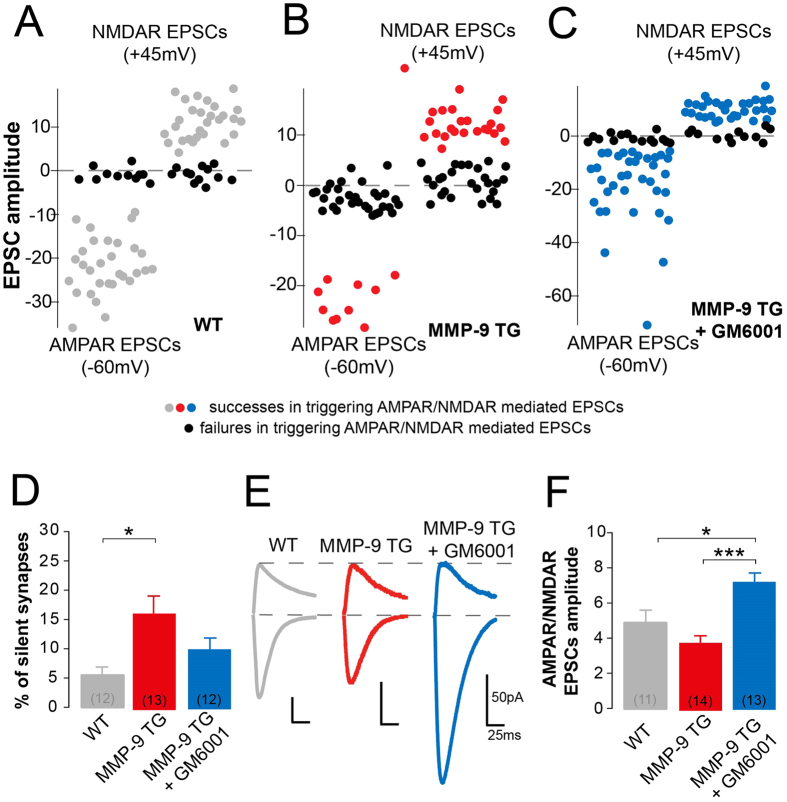
Inhibition of MMP-9 activity reduces the number of AMPAR-silent synapses presumably through the recruitment of AMPARs to the synapse. (**A–C**) Schematic representation of the minimal stimulation protocol that was used to calculate the number of silent synapses. The graphs show 40–50 consecutive stimulation trials, resulting in either successes (gray, blue or red circles) or failures (black circles) in triggering AMPAR- and NMDAR-mediated EPSCs, recorded at −60 mV and +45 mV, respectively. (**D**) Summary bar graph that shows an increase in the number of silent synapses in rats that overexpressed MMP-9. Application of the MMP inhibitor GM6001 (25 μM) decreased the number of AMPAR-silent synapses. Numbers in brackets represent the number of recorded cells. (**E**) Example average traces of AMPA EPSCs that were recorded at −60 mV and composite AMPA and NMDA EPSCs that were recorded at +45 mV, peak-scaled to the size of the NMDA response in WT rats (shown in gray). The relative amplitude of AMPA responses decreased in MMP-9 TG animals (red) and increased upon MMP-9 inhibition with GM6001 (blue). (**F**) Summary bar graph that shows a decrease in the ratio of AMPA/NMDA EPSCs amplitudes in rats that overexpressed MMP-9. Application of the MMP inhibitor GM6001 increased the relative size of AMPAR-mediated currents. Numbers in brackets represent the number of recorded cells.

Silent synapses are frequently located on long, thin spines. Such synapses have been shown to exhibit a low AMPAR/*N*-methyl-D-aspartate (NMDA) receptors ratio that increases when synapses mature and incorporate more AMPARs^26^. To determine whether changes in the morphology of dendritic spines that is driven by MMP-9 activity and its inhibition correlate with silent synapses and AMPAR/NMDAR ratio (Fig. 3A–C), we determined the number of silent synapses in the CA1 area of the hippocampus in MMP-9 TG and wild type rats. We first tested whether an increase in MMP-9 activity correlates with the number of silent synapses. We found that the percentage of silent synapses was significantly higher in slices from MMP-9 TG rats (16.23% ± 3.72%), compared with slices from wild type animals (5.47% ± 1.7%; Fig. 3D). Thus, chronic, excessive MMP-9 activity increases the abundance of silent synapses in the CA1 area of the hippocampus. We then determined whether this elevated level of silent synapses could be reduced by inhibiting MMP-9 activity in slices from MMP-9 TG rats. Treatment with the general inhibitor GM6001 resulted in an approximately 30% reduction of silent synapses in MMP-9 TG rats (Fig. 3D). Although, this effect was not statistically significant, as compared with slices from untreated MMP-9 TG rats, the number of silent synapses did not significantly differ between wild type and TG inhibitor-treated slices. To investigate the effect of inhibiting AMPAR recruitment, we determined the ratio of AMPA/NMDA receptors (Fig. 3E,F). This parameter informs about changes in a neuron’s sensitivity to glutamate and can be modified by the insertion or removal of AMPARs from the synapse. Indeed, in slices from MMP-9 TG rats that were treated with GM6001, the AMPAR/NMDAR ratio significantly increased in comparison with MMP-9 TG rats that were not treated with the inhibitor and wild type animals. These results suggest that MMP-9 hyperactivity promotes the silencing of synapses, which can be reversed by MMP-9 inhibition. This result correlated with changes in the number of silent synapses, further supporting the notion that MMP-9 inactivation triggers the incorporation of new AMPARs to the synapse.

### Long-term potentiation in TG rats that manifests excessive proteolytic activity is altered and can be rescued by application of the protease inhibitor

**Figure 4:**
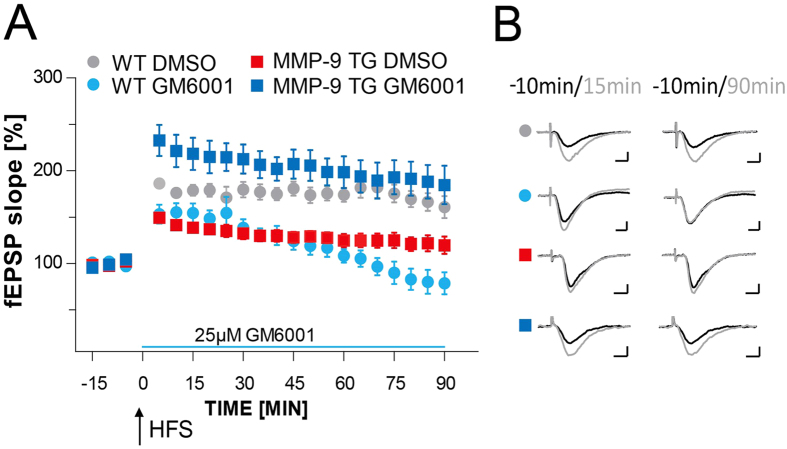
Long-term potentiation in TG rats that overexpress MMP-9 is altered and can be rescued by applying an MMP inhibitor. **(A)** The figure shows the time course of maximal EPSP slopes normalized to baseline in the CA1 region of the hippocampus. Long-term potentiation that was induced by high-frequency stimulation (HFS; 3× 100 Hz; black arrow) of the Schaffer collaterals in the presence of DMSO in slices from MMP-9 TG rats had a lower magnitude (filled red squares; *n*_rats_ = 6; 130.8% ± 6.2% of baseline) compared with control slices from WT rats (filled gray circles; *n*_rats_ = 6; 175.7% ± 8.4% of baseline; *p* = 0.0004). The inhibitor of metalloproteinases, GM6001, was applied immediately after HFS and improved LTP that was evoked in slices from MMP-9 TG rats (filled dark blue squares; *n*_rats_ = 6; 203.1% ± 16.8% of baseline) compared with slices from the same animals that were treated with the solvent of inhibitor only (filled red squares; *p* = 0.001). GM6001 application during the recordings from slices that were obtained from WT rats caused LTP destabilization 30 min after LTP induction (filled light blue circles; *p* = 0.002, compared with DMSO-treated slices from WT rats; filled gray circles). The light blue line marks the time when GM6001 was present in the experimental solution. Error bars represent the SEM. **(B)** Representative traces of fEPSP 10 min before (black) and 15 and 90 min after (gray) the induction of LTP are shown. Scale bars = 2 mV and 5 ms.

To determine whether excessive MMP-9 activity that is caused by enzyme overexpression affects synaptic plasticity, we performed a series of *in vitro* extracellular recording experiments using hippocampal slices from transgenic MMP-9 rats. Tetanic stimulation (3× 100 Hz, 1 s duration) evoked stable LTP, lasting at least 90 min, in both MMP-9 TG rats and control animals (Fig. 4). However, LTP in MMP-9 TG rats was consistently lower as compared with control wild type rats.

To determine whether the diminished LTP that was observed in slices from MMP-9 TG rats was caused by MMP-9 hyperactivity, we investigated whether LTP could be rescued by inhibiting proteolytic activity. We performed LTP experiments in slices from transgenic animals in the presence of GM6001 that was administered immediately after the last train of 100 Hz stimulation. Long-term potentiation that was evoked in slices from MMP-9 TG rats in the presence of GM6001 was significantly higher compared with untreated slices from the same animals (Fig. 4). We also used GM6001 while recording in slices from control animals. Long-term potentiation that was evoked in slices that were treated with the inhibitor according to the same protocol was stable during the first 30 min and then declined to baseline levels thereafter. Long-term potentiation in slices that were treated only with DMSO was stable throughout the entire recording session (Fig. 4). Basal synaptic transmission defined as input-output ratio was not statistically different between MMP-9 TG and wild type rats.

Previous studies showed that MMP-9 inhibition resulted in the disruption of L-LTP^15^,^27^. Therefore, the results of the present experiments are surprising, as they demonstrate that MMP inhibition may enhance LTP, provided that the inhibitors are applied under conditions of initially excessive MMP-9 activity.

### Inhibition of endogenous MMP-9 is required for the maturation of dendritic spines *in vitro*

**Figure 5:**
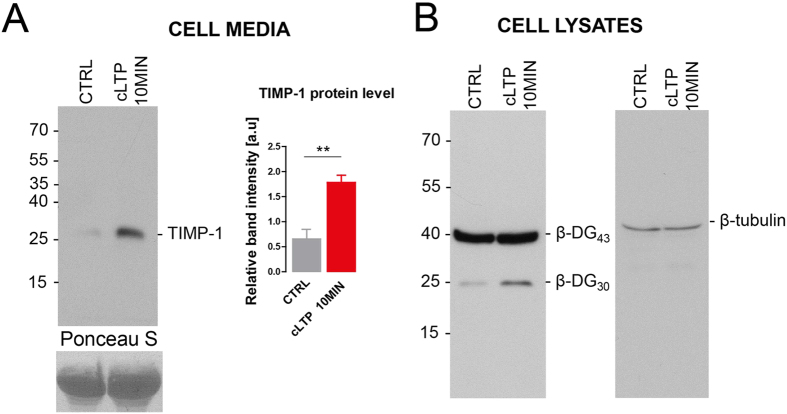
cLTP elevates TIMP-1 protein expression (**A)** Representative Western blot on conditioned media obtained from 21 DIV dissociated hippocampal cultures that were stimulated with cLTP. An increase in the level of TIMP-1 protein occurred 10 min after cLTP (1.78 ± 0.12; *p* = 0.008) stimulation compared with control media that were treated with DMSO (0.65 ± 0.14). (**B**) Representative Western blot of cell lysates from 21 DIV dissociated hippocampal cultures. Enhanced proteolysis of β-dystroglycan occurred 10 min after cLTP stimulation compared with the control (DMSO).

TIMP-1 is a well-established endogenous inhibitor with specific affinity for MMP-9^28^. Thus, we decided to investigate its role in the synaptic plasticity. As the model system, we have employed chemical LTP (cLTP) stimulation of neuronal cultures, since they are relatively easy to manipulate and MMP-9 has previously been linked to this kind of synaptic plasticity^16^,^29^. First, we determined whether TIMP-1 expression was elevated after cLTP stimulation, by examining the amount of protein using Western blot. Previous studies showed that treatment with rolipram, forskolin, and picrotoxin produces lasting (up to several hours) enhancement of network activity, leading to changes in dendritic spine shape^12^,^20^. From the study by Szepesi *et al*.^29^ we also know that the level of MMP-9 protein is increased 10 min after cLTP stimulation. In the present experiment, we observed a marked increase in TIMP-1 protein amount (Fig. 5A) in the media of cLTP-stimulated cells compared with the media of control cells that were treated with the solvent of cLTP components (DMSO). We also determined the efficacy of cLTP stimulation-evoked MMP-9 activity on cell lysates. The Western blot analysis revealed an increase in the level of the cleaved substrate of MMP-9 (β-dystroglycan)^18^ in cLTP-treated samples (Fig. 5B). The fact that cLTP induces β-dystroglycan cleavage was reported previously^18^,^30^.

**Figure 6:**
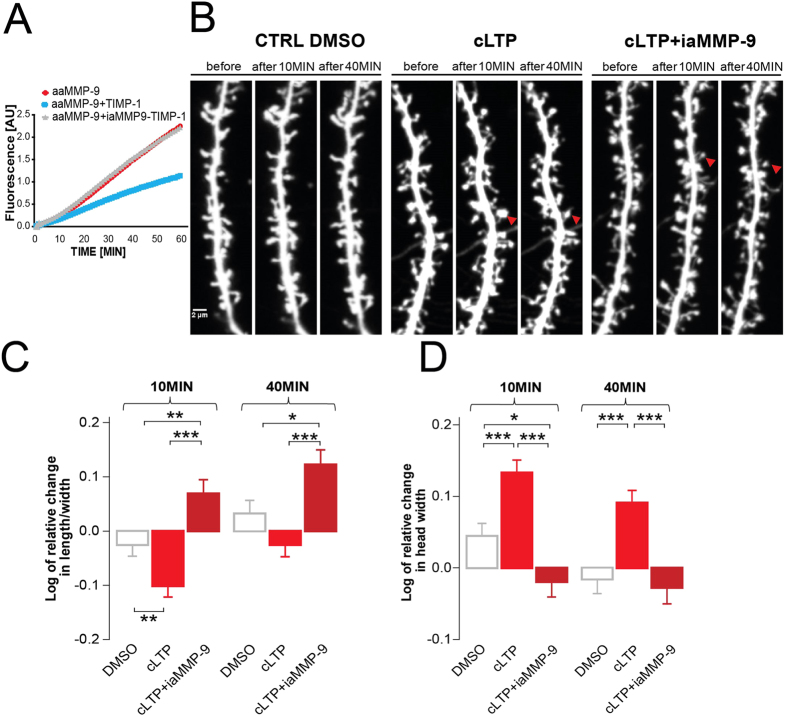
Inhibition of endogenous MMP-9 is required for the maturation of dendritic spines in dissociated hippocampal cultures. **(A)** Results of the DQ-gelatin assay. MMP-9 activity (red) was inhibited by TIMP-1 (blue). The effect of MMP-9 inhibition by TIMP-1 was blocked by incubation with iaMMP-9 (gray). **(B)** Representative images of live cell imaging of 21 DIV hippocampal neurons that expressed EGFP, revealing changes in dendritic spine morphology (indicated by red arrows) after incubation with cLTP+iaMMP-9. The same set of dendritic spines was analyzed before and after stimulation. **(C)** Relative changes (mean ± SEM) in the length/width ratio of dendritic spines incubated with cLTP+iaMMP-9. The spines became longer and thinner after 10 min cLTP+iaMMP-9 treatment (*n*_spine_ = 370; length/width, 0.069 ± 0.026; *p* < 0.001 and *p* = 0.004) compared with stimulation without sequestration of the endogenous inhibitor (cLTP only; *n*_spine_ = 641; length/width, −0.101 ± 0.021) and control (DMSO, vehicle of cLTP; *n*_spine_ = 379; length/width, −0.025 ± 0.021). After 40 min, the changes in dendritic spine length were persistent. cLTP+iaMMP-9 stimulation increased the length/width ratio (*n*_spine_ = 370; length/width, 0.120 ± 0.027; *p* < 0.001 and *p* = 0.01) compared with cLTP (*n*_spine_ = 641; length/width, −0.253 ± 0.022) and the control (*n*_spine_ = 379; length/width, 0.032 ± 0.024). **(D)** Relative changes (mean ± SEM) in the head width of dendritic spines. Dendritic spine heads after 10 min cLTP were wider (*n*_spine_ = 641; head width, 0.133 ± 0.018; *p* < 0.001 and *p* < 0.001) compared with dendritic spines in cLTP+iaMMP-9 treatment (*n*_spine_ = 370; head width, −0.019 ± 0.021) and control (*n*_spine_ = 364; head width, 0.044 ± 0.018). Stimulation for 40 min with cLTP induced enlargement of head width (*n*_spine_ = 641; head width, 0.091 ± 0.017; *p* < 0.001 and *p* < 0.001) compared with cLTP+iaMMP-9 (*n*_spine_ = 370; head width, −0.027 ± 0.023) and the control (*n*_spine_ = 364; head width, −0.016 ± 0.020).

To determine whether inhibiting of endogenously excessive MMP-9 activity is required for the maturation of dendritic spines, we sequestered TIMP-1 by the use of (inactive mutant of MMP-9) iaMMP-9^23^. Such an approach to blocking TIMP-1 was previously proposed^31^, and its efficacy in an enzymatic assay is shown in Fig. 6A.

The detailed analysis of changes in the shape of dendritic spines showed that when iaMMP-9 scavenged endogenous TIMP-1, dendritic spines did not become mushroom-shaped after cLTP and remained elongated, similarly to conditions of excessive exogenous MMP-9 activity (see above). Figure 6B shows representative images of the same segments of dendrites before and after treatment. The quantitative analysis of the length/width parameter that was measured in individual dendritic spines is shown in Fig. 6C. Ten minutes of cLTP stimulation with iaMMP-9 application significantly increased the length/width parameter as compared with cLTP-stimulated cells. Compared with control neurons the cells that were treated with cLTP+iaMMP-9 exhibited a significant increase in the length/width ratio (Fig. 6C). cLTP stimulation itself also caused changes in the shape of dendritic spines toward shorter and wider spines. Further analysis showed that these effects were persistent. Forty minutes of cLTP stimulation with iaMMP-9 application also increased the length/width ratio of dendritic spines compared with cLTP stimulation alone. cLTP stimulation for 40 min did not significantly influence the length/width parameter of dendritic spines.

To support our results that suggested that cLTP increases the volume of dendritic spine heads, we also studied the relative changes in the width of dendritic spine heads following treatment (Fig. 6D). The analysis of dendritic spine morphology showed that head width was significantly increased by cLTP stimulation. In contrast, head width was significantly lower in neurons that were treated with cLTP+iaMMP-9 (Fig. 6D). The increase in spine head volume was persistent and lasted until the end of observation.

Based on these experiments, we conclude that the inhibition of endogenous MMP-9 activity by TIMP-1 is required for the maturation of dendritic spines during enhanced neuronal activity.

### Inhibition of endogenous MMP-9 is required for LTP maintenance

**Figure 7:**
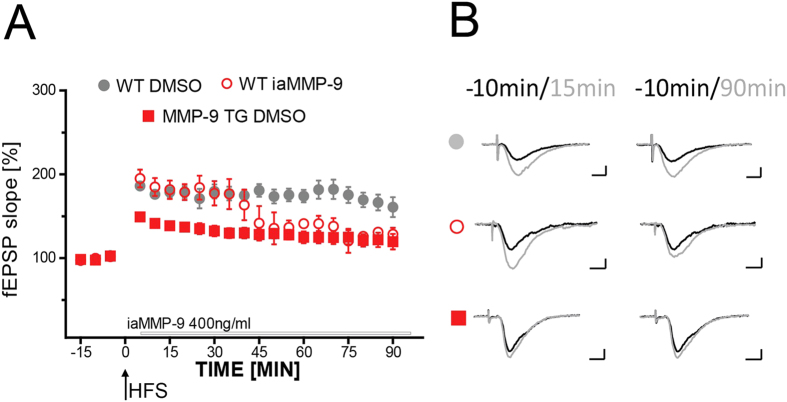
Inhibition of endogenous MMP-9 is required for LTP maintenance. **(A)** The figure shows the time course of maximal EPSP slopes normalized to baseline measured in the CA1 area of the hippocampus. High-frequency stimulation in slices from WT rats that were treated with iaMMP-9 (open red circles; *n*_rats_ = 5) evoked LTP at a similar level (180.2% ± 11.5% of baseline) as in untreated slices from wild type rats (filled gray circles; *n*_rats_ = 6; *p* = 0.560 for the first 45 min after LTP induction). After approximately 45 min, LTP on slices from WT rats treated with iaMMP-9 declined to the level that was previously observed in slices from MMP-9 TG rats that were treated with DMSO (filled red squares; *n*_rats_ = 6; *p* = 0.026, compared with untreated slices from wild type animals for the last 45 min of recording; *p* = 0.358, compared with untreated slices from MMP-9 TG rats). Black arrows mark the time of application of HFS. The open gray line marks the time when iaMMP-9 was present in the experimental solution. Error bars represent the SEM. **(B)** Representative traces of fEPSP 10 min before (black) and 15 and 90 min after (gray) LTP induction are shown. Scale bars = 2 mV and 5 ms.

In the last set of experiments we investigated effects of abrogating inhibitory activity of TIMP-1 on LTP in the hippocampal slices. We applied iaMMP-9 to slices from control wild type rats to block TIMP-1 function after high-frequency stimulation (HFS). iaMMP-9 was applied after the last train of 100 Hz stimulation and did not interfere with the magnitude of LTP within the following 40 min. After that time, LTP magnitude declined to the level that was observed previously in slices from MMP-9 TG rats (Fig. 7).

These experiments demonstrated that the endogenous inhibition of MMP-9 controlled the proper neuronal response to stimulation. MMP-9 activity is crucial for LTP maintenance, but its overabundance that is caused by TIMP-1 sequestration is detrimental to this form of plasticity.

## Discussion

**Figure 8:**
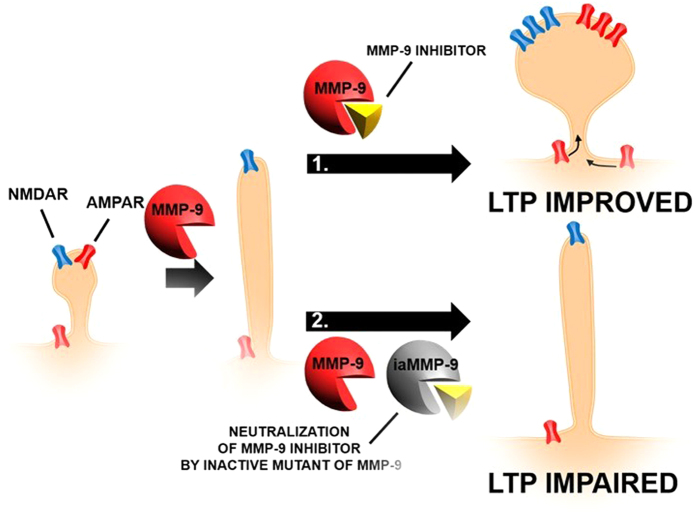
Transient ECM protease activity promotes synaptic plasticity. Proteolytic activity *per se* initiates the promotion of structural and functional plasticity, which requires subsequent endogenous enzymatic inhibition to be concluded. Thus, spines first become elongated based on MMP activity. Subsequently, extracellular proteolysis is terminated (due to TIMP-1), resulting in dendritic spine growth that is expressed as expansion of its head and the incorporation of AMPARs to previously silent synapses, a process that is required for LTP maintenance.

In the present study, we have found that plastic changes in synaptic structure and function can be a consequence of transient, strictly controlled proteolysis at the synapse (Fig. 8). Proteolytic activity *per se* initiates the promotion of structural and functional plasticity, which, to be concluded, requires subsequent endogenous enzymatic inhibition. Thus, dendritic spines first become elongated due to MMP activity and subsequently, extracellular proteolysis is terminated (due to the endogenous inhibitor of MMP), resulting in dendritic spine growth that is expressed as expansion of its head. This phenomenon may involve incorporation of AMPARs to previously silent synapses, a process that is required for LTP maintenance.

In the experiments described herein, first, exogenous MMP-9 was used to induce morphological changes in dendritic spines, and the enzyme was subsequently blocked with a chemical inhibitor. Second, in a model of synaptic plasticity produced in neuronal culture (chemical LTP, cLTP) where enhancement of endogenous MMP-9 at the synapses was described^12^,^32^,the inhibitory activity of TIMP-1 was switched off using a novel approach with a recombinant inactive mutant of MMP-9, which scavenges the endogenous inhibitor. The effects of these manipulations on the shape of dendritic spines were determined, showing that the spines first elongate through an MMP-9-dependent process and then mature to produce larger spine heads after turning off MMP-9 activity. We also report that hippocampal slices from transgenic rats that overexpress MMP-9 are impaired in electrically-evoked LTP, the effect that could be rescued by inhibiting excessive MMP-9 activity. Furthermore, we demonstrate that changes in dendritic spine morphology correspond to AMPAR content.

Recently, Tsien^14^ proposed that very long-term memories, such as those resulting from fear conditioning, are stored as a pattern of holes in the perineuronal net (PNN), a specialized ECM that envelops mature neurons and restricts synapse formation. Furthermore, he suggested that the proteolytic activity of MMP-9 may be responsible for the generation of such holes. Therefore, one can infer that unrestricted proteolytic activity should promote the development of larger, more efficacious dendritic spines. However, our findings suggest, somewhat counterintuitively, that the precisely controlled termination of MMP-9-driven proteolysis is mandatory for spine growth and functional maturation.

We showed that the enzymatic activity of MMP-9 promotes the elongation of dendritic spines in both dissociated hippocampal cultures and MMP-9 TG rats. These observations are consistent with our previous study^23^. The novel, and at a first sight paradoxical observation presented herein is that the subsequent inhibition of MMP-9 activity results in the termination of dendritic spine elongation and leads to changes in spine morphology toward a mushroom shape with increased efficacy. The data presented show that elongated spines may mature, following the inhibition of enzyme activity.

We also suggest that elongated dendritic spines in MMP-9 TG animals form silent synapses, and the inhibition of MMP-9 activity transforms the synapses to mature forms both functionally and morphologically. We show here that after the inhibition of excessive MMP-9 activity in slices of TG rats, the number and/or function of AMPAR increases significantly as it is shown by the increase in AMPAR/NMDAR ratio. The increase in AMPA receptors content accompanies unsilencing of the silent synapses. The changes in the number of silent synapses after the inhibition of MMP-9 are subtle. However, they can be explained by the decrease in lateral diffusion of GluA1 type of AMPAR at the synapses, that was demonstrated in cLTP in our previous study^12^. The relationship between spine morphology and the expression of functional AMPARs has been well documented^12^,^33^. Small and thin structures were shown to have limited sensitivity to glutamate^33^, which appeared to be a consequence of low levels of AMPARs^34^,^35^. Notably, previous findings have shown that MMP-9 protein is predominantly located on small spines^36^ that mature during cLTP in an MMP-9-dependent manner, and this enlargement is strictly correlated with AMPAR recruitment and immobilization^12^.

Previous studies repeatedly showed that MMP-9 inhibition precludes the maintenance of LTP and impairs long-term memory^10^,^15^,^16^,^27^,^36^,^37^. In the present study, we confirmed that the incubation of hippocampal slices from wild type rodents with an MMP inhibitor that prevents MMP-9 activation impaired the maintenance of LTP without changing either LTP induction or synaptic transmission as reported in the previous experiments demonstrating similar results on MMP-9 knockout or following the application of inhibitors before learning/LTP onset. Herein, we found that excessive MMP-9 activity in TG rats impaired both the induction and maintenance of LTP. Surprisingly, this impairment of the synaptic response could be rescued and returned to levels that were observed in slices from wild type rats by subsequently inhibiting MMP activity. One can raise an issue that there is a rundown effect observed due to MMP inhibitor application in LTP experiment. However, it is common for recording in the presence of MMP inhibitors, as it was observed previously^9^,^27^. Of importance, in our experiments such an effect does not influence stability of LTP recording independently on time of signal integration.

Although previous studies showed the detrimental effect of exogenous TIMP-1 on neuronal plasticity^10^,^38^ and other studies strongly supported the idea that MMP inhibition is functionally involved in synaptic plasticity^39^,^40^,^41^,^42^,^43^,^44^ the precise mechanism of this process in the structural and functional remodeling of synapses remained unknown. It is known from previous study that 5 minutes after synaptic stimulation there is an increase in MMP-9 activity^18^. Additionally, it was shown previously that mRNA of TIMP-1 was upregulated after LTP^45^. Our novel data demonstrated that inhibition, provided within minutes after such onset (i.e., after MMP-9 was already activated), was capable of promoting synaptic plasticity, which may appear paradoxical, but can be explained by the simultaneous secretion of MMP and its inhibitor. Sbai *et al*.^46^ have shown that MMPs and TIMP-1 are secreted in 160–200 nm vesicles in a Golgi-dependent pathway. These vesicles distribute along microtubules and microfilaments, colocalize differentially with the molecular motors kinesin and myosin Va and undergo both anterograde and retrograde trafficking. MMP-9 vesicles are preferentially distributed in the somato-dendritic compartment and are found in dendritic spines^46^.

We found that the inhibition of endogenously induced MMP-9 hyperactivity is pivotal for proper synaptic structure and synaptic responsiveness. We induced the endogenous activity of MMP-9 by applying stimulation that produced robust and steady synaptic enhancement (cLTP or LTP). Both chemical and electrophysiological LTP was shown to induce the maturation of dendritic spines in an MMP-9-dependent manner^11^,^12^. In the present study, we found that the endogenous inhibition of MMP-9 contributed to the morphological changes in dendritic spines during synaptic potentiation. TIMPs tightly bind to the active forms of MMPs forming 1:1 complexes, thus blocking their activity^47^. To interfere with MMP/TIMP system we applied an inactive mutant of MMP-9 (iaMMP-9) that used in excess may exhibit high affinity for TIMP-1^48^. iaMMP-9 can bind also other TIMPs especially to TIMP-3^28^. However, we show here an importance of the cooperation of the protease and its inhibitor, in this case the interaction of iaMMP-9 with other inhibitor can even improve the observed effects. TIMP-1 sequestration prevented dendritic spines from maturing. Head width (i.e., the parameter that corresponds to the accumulation AMPARs at a synapse^34^,^35^) significantly decreased after stimulating the neurons while simultaneously sequestering TIMP-1. Furthermore, LTP that was induced in hippocampal slices from wild type animals with simultaneous sequestration of TIMP-1 declined after 30 min to the level of MMP-9 TG rats, demonstrating that MMP-9 inhibition is required for dendritic spine maturation and LTP maintenance.

Incidentally, our study may explain the observed therapeutic effect of MMP-9 inhibition in Fragile X syndrome. Fragile X syndrome has been shown to be associated with markedly enhanced MMP-9 activity^24^,^49^,^50^. The inhibition of MMP-9 activity (e.g., with minocycline) may be beneficial in both animal models and humans^24^,^51^,^52^,^53^,^54^. Our data suggest that an increased level of MMP-9 that is caused by changes in local translation^49^,^50^ in Fragile X syndrome is excessively high and its endogenous inhibitor is not able to control it. Such an imbalance between this protease and its inhibitor may lead to abnormalities in dendritic spine morphology that, in turn, are responsible for behavioral symptoms. Treatment with an MMP-9 inhibitor may restore a proper balance between MMP-9 and its inhibitor, with rescue of the morphology of spines and behavioral improvements.

Finally, our results may also provide an explanation for the contradictory effects of MMP-9 on dendritic spines that have been reported in previous studies^55^. The activity of MMP-9 was shown to lead to the elongation of dendritic spines^20^,^23^,^24^. In contrast, other studies demonstrated that MMP-9 activity caused the maturation of dendritic spines^11^,^50^. In the present study, we found that the control of MMP-9 activity by the endogenous inhibitor is responsible for the maturation of spines.

In conclusion, the present results show that proteolysis at the synapse is strictly controlled endogenously, and the inhibition of proteolysis affects the efficacy of the synaptic response. Alterations in the fine balance between the protease and its inhibitor may be suggested to lead to detrimental effects and result in neuropsychiatric diseases.

## Materials and Methods

### Recombinant autoactivating MMP-9 and inactive MMP-9

Expression of the previously described^56^ auto-activating mutant of MMP-9 was performed using the Bac-to-Bac Baculovirus expression system, according to the manufacturer’s instructions (Invitrogen). Briefly, the MMP-9 G100L mutant (a gift from Katherine Fisher, Pfizer, Groton, PA) was cloned into pFastBac1, and the resulting recombinant plasmid was used to transform DH10Bac competent cells. Colonies that performed transposition of recombinant plasmid fragment into bacmid DNA were identified by blue–white selection, and recombinant bacmid was isolated and verified by PCR. The Sf21 insect cells were transfected with recombinant bacmid using Cellfectin reagent (Invitrogen) to obtain recombinant baculovirus. After amplification and titration of the recombinant baculovirus, High-Five cells were infected and incubated in the Sf-900IISFM serum-free medium (Invitrogen). Conditioned medium was collected for analysis of expression by gel zymography. At 24, 48 and 72 hours after infection, medium was collected and an equal volume was tested by gel zymography. Thus, the cell medium harvested 48 hours after the infection with baculovirus was used for purification of recombinant autoactivating mutant of MMP-9 by affinity chromatography with gelatin–Sepharose 4B (GE Healthcare) as previously described^57^. Protein concentrations in the collected fractions were measured using Bradford reagent (Sigma). The recombinant inactive mutant MMP-9 (E402A) was generated using QuikChange (Stratagene) according to the manufacturer’s instructions. The point mutation changing glutamate 402 into alanine in the catalytic centre of human MMP-9 was inserted by PCR using a pair of primers: 5′-TGGCGGCGCATGCGTTCGGCCACGC-3′ and 5′-GCGTGGCCGAACGCATGCGCCGCCA-3′. Next MMP-9 E402A was cloned into the pFastBac1 vector, and expression and purification of protein was performed exactly as described for MMP-9. The enzymatic activity of purified MMP-9 and MMP-9 E402A was checked with the EnzCheck gelatinase/collagenase assay kit (Invitrogen) according to the manufacturer’s instructions.

### Electrophysiology

This study was carried out in accordance with the Ethical Committee on Animal Research of the Nencki Institute, based on the Polish Act on Animal Welfare and other national laws that are in full agreement with EU directive on animal experimentation. The protocols were approved by the Committee on the Ethics of Animal Experiments of the Nencki Institute (Permit Number: 454/2013). All effort was made to minimize animal suffering.

Transgenic male rats that overexpressed aaMMP-9 under the control of the synapsin-1 promoter (MMP-9 TG) and their wild type male littermates were used for both extracellular recordings and dendritic spine morphology analysis. At 2.5–3.5 months of age, the rats were anesthetized with isoflurane and decapitated. The brains were quickly removed and placed in cold artificial cerebrospinal fluid (aCSF; 117 mM NaCl, 1.2 mM MgSO_4_, 4.7 mM KCl, 2.5 mM CaCl_2_, 25 mM NaHCO_3_, 1.2 mM NaH_2_PO_4_, and 10 mM glucose, bubbled with 95% O_2_/5% CO_2_). Both hemispheres were cut into 400 μm coronal slices with a vibratome (Leica VT 1000S). Slices that contained the hippocampus were placed in a recording interface chamber (Harvard Apparatus) to recover for at least 1.5 h before recordings began and continuously perfused with carbonated aCSF at 33 ± 1 °C. Field excitatory postsynaptic potentials (fEPSPs) were recorded from the stratum radiatum area of the CA1 field using a glass pipette that was filled with 1 M NaCl (impendence: 1.0–3.0 MΩ). fEPSPs were evoked by the stimulation of the Schaffer collateral-commissural every 30 s (test pulses at 0.033 Hz, 0.1 ms) using bipolar metal electrodes (FHC). The intensity of the test stimulus was adjusted to obtain fEPSPs with slopes that were one-third of the maximal response. After at least 15 min of stable baseline, LTP was tetanically induced (three trains of 100 Hz, 1 s stimulation, separated by 3 min). After the end of the stimulation protocol, a test pulse was subsequently applied for at least 90 min. Recordings were amplified (EX4-400 Dagan Corporation) and digitized (POWER 1401, CED), and the fEPSP slopes were analyzed online and offline using SIGNAL software (CED). For the analysis of LTP, the response slopes were expressed as a percentage of the average response slopes during the baseline period prior to LTP induction. Bath-applied reagents included the following: GM6001 (25 μm; Millipore) and recombinant iaMMP-9 (400 ng/ml), dissolved in aCSF, were administered immediately after tetanic stimulation, and present in aCSF throughout the remainder of the experiment. Control experiments and baseline recordings for experiments with GM6001 or recombinant iaMMP-9 were conducted in the presence of DMSO or the buffer in which recombinant iaMMP-9 was dissolved (50 mM Tris-HCl [pH 7.5], 400 mM NaCl, 10 mM CaCl_2_, and 2% DMSO), respectively (final concentration of DMSO was lower than 0.02%). All LTP experiments were performed in the same way and repeated three times. Statistical analyses between groups were performed using repeated-measures analysis of variance (ANOVA) and Statistica 8.0 software (StatSoft). Values of *p* < 0.05 were considered statistically significant.

Slices for silent synapse calculations were prepared similarly to the methods described above. They were cut into 300 μm thick slices and allowed to rest for 30 min before the recordings. The recording chamber was perfused with an aCSF solution that additionally contained 50 μM picrotoxin (Sigma-Aldrich) (for all of the experiments) and 25 μM GM6001 and 14 mM DMSO (for the specified recordings), heated to 29–31 °C, and constantly bubbled with carbogene. CA1 pyramidal neurons were visually identified and patched with a borosilicate glass pipette (3–5 MΩ resistance). The patch pipette was filled with internal solution: 130 mM Cs gluconate, 20 mM HEPES, 3 mM TEA-Cl, 0.4 mM EGTA, 4 mM Na_2_ATP, 0.3 mM NaGTP, and 4 mM QX-314Cl. The pH was adjusted to 7.0–7.3 using CsOH, and osmolarity was adjusted to 290–295 mOsm with CsCl. Recordings were performed in whole-cell voltage clamp mode and filtered at 2 kHz. Series and input resistances were monitored throughout the experiment.

The electrical stimulation of Schaffer collaterals was performed using a bipolar AgCl electrode. A single electrical stimulus with a 0.2 ms duration was applied every 5 s. For the AMPAR/NMDAR ratio analysis, 30–50 stable sweeps were recorded at −60 mV and +45 mV for AMPA and NMDA excitatory postsynaptic currents (EPSCs), respectively, and their peak amplitudes were averaged. The amplitude of NMDAR-mediated EPSCs was measured 60 ms after the peak response to ensure the absence of the AMPAR component.

For silent synapse calculations, a minimal stimulation protocol was used, in which the electrical stimulus was decreased to obtain both successes and failures of AMPA and NMDA responses (failures *vs*. successes were defined visually). The percentage of silent synapses was then calculated based on the following equation:


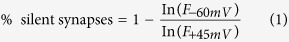


where *F*_*−60mV*_ and *F*_*+45mV*_ are failure rates at −60 and +45 mV, respectively. The statistical analysis was performed using the Tukey *post hoc* test.

### DiI staining of hippocampal slices

For the visualization of changes in the shape of dendritic spines, of 1,1′-Dioctadecyl-3,3,3′,3′-Tetramethylindocarbocyanine Perchlorate (DiI) staining in MMP-9 TG and wild type animals was performed. The brains were prepared as described above for the electrophysiological analysis. Slices (300 μm) were then cut on a vibratome (Leica VT 1000S). Slices that contained the hippocampus were placed in tubes (Harvard Apparatus) and continuously perfused with carbonated aCSF at 33 ± 1 °C to recover for at least 1.5 h. Bath application of GM6001 (25 μm) or DMSO (control) was then performed on the slices for 90 min. After treatment, the slices were fixed with 4% paraformaldehyde in phosphate-buffered saline (PBS; 30 min at 4 °C) and kept in cold PBS until further procedures were performed. Random dendrite labeling was performed using 1.6 μm tungsten particles (Bio-Rad, Hercules, CA, USA) that were coated with propelled lipophilic fluorescent dye (DiI; Invitrogen) that was delivered to the cells by gene gun (Bio-Rad) bombardment. Images of dendrites (50–200 μm) from the cell soma of the CA1 field of the hippocampus were acquired under 561 nm fluorescent illumination using a confocal microscope (63× objective, 1.4 NA) at a pixel resolution of 1024 × 1024 with a 3.43 zoom, resulting in a 0.07 μm pixel size. The average diameter of analyzed dendritic branches was between 0.69 to 0.78 μm. Thus the variation in the size of dendritic branches was less than 15%.

### Western blot

Hippocampal neurons at 18–21 DIV were lysed after stimulation in lysis buffer that contained 20 mM Tris-Cl (pH 6.8) at 4 °C, 137 mM NaCl, 25 mM β-glycerophosphate, 2 mM NaPPi, 2 mM EDTA, 1 mM Na_3_VO_4_, 1% Triton X-100, 10% glycerol, 2 mM benzamidine, 1 mM phenylmethylsulfonyl fluoride, 0.5 mM dithiothreitol, and 10 μl/ml protease inhibitor mixture (Sigma-Aldrich). Samples of culture media and lysates that contained equal amount of protein were mixed with 4× sodium dodecyl sulfate (SDS) sample buffer and denaturated by heating at 95 °C. The samples (20 μg) were subjected to 12% SDS-polyacrylamide gel electrophoresis and then electrotransferred onto polyvinylidene difluoride membranes (Immobilon-P, Millipore). Ponceau S staining was performed to locate the protein bands on Western blots. The membranes were then blocked in 10% nonfat milk in Tris-buffered saline with 0.1% Tween 20 (TBST) and incubated at 4 °C overnight with anti-β-dystroglycan (1:500; NCL-b-DG, Novocastra), anti-β-tubulin (1:1000; ab21058, Abcam), and anti-TIMP-1 (1:500; MAB580, R&D System) diluted in 10% nonfat milk in TBST. The membranes were then incubated with peroxidase-labeled secondary antibodies diluted 1:10000 in 10% nonfat milk in TBST for 1 h at room temperature. ECL Plus reagent (GE Healthcare) was used to detect horseradish peroxidase (HRP) on the immunoblots.

### Dye-quenched gelatin assay

To test whether iaMMP-9 is able to bind TIMP-1 and block its inhibitory effects on the enzymatic activity of MMP-9, we used the EnzChek gelatinase/collagenase assay kit (Molecular Probes). Dye-quenched gelatin (DQ-gelatin) is a substrate for MMP-9. It is heavily labeled with FITC so that fluorescence is quenched until the gelatin is cleaved. The assay was performed in a 96-well Macro-assay plate (96-well; Greiner Bio-one). The procedure was performed according to the manufacturer’s instructions. aaMMP-9 (400 ng/ml) was incubated alone (positive control) or with TIMP-1 (500 ng/ml) or a mixture of TIMP-1 and iaMMP-9 (400 ng/ml) in reaction buffer with DQ-gelatin. The plate was then placed in a fluorescence reader (Infinite M200, Tecan) and fluorescence was measured every minute for 1 h at 37 °C (excitation wavelength, 495 nm; emission wavelength, 515 nm). The kinetics of the enzymatic reactions were corrected for background by subtracting the non-enzymatic control. The graphs were generated and the calculations were performed using Prism 6 software (GraphPad Software).

### Dissociated hippocampal cultures

Dissociated hippocampal cultures were prepared from postnatal day 0 (P0) Wistar rats. Brains were removed, and hippocampi were dissected on ice in dissociation medium (DM; 81.8 mM Na_2_SO_4_, 30 mM K_2_SO_4_, 5.8 mM MgCl_2_, 0.25 mM CaCl_2_, 1 mM HEPES [pH 7.4], 20 mM glucose, 1 mM kynurenic acid, and 0.001% phenol red). The hippocampi were then rinsed in DM, incubated twice with papain solution (100 U in DM) for 15 min at 37 °C, and subsequently rinsed three times in DM. Papain activity was stopped by applying a trypsin inhibitor dissolved in DM. The hippocampi were then washed three times in plating medium (PM; Minimum Essential Media; 10% fetal bovine serum; 1% penicillin-streptomycin) and triturated until the medium became cloudy. Triturated hippocampi were then diluted 10 times in PM and centrifuged for 10 min at 1000 × *g* at room temperature. Cell pellets were suspended in PM, and cells were counted and plated at a density 90,000 cells per 18-mm-diameter coverslip that was coated with 1 mg/ml poly-D-lysine (Sigma-Aldrich) and 2.5 μg/ml laminin (Roche). Two hours after plating, the PM was exchanged for maintenance medium (MM; Neurobasal-A, 2% B-27 supplement, and 1% penicillin-streptomycin). The cells were maintained at 37 °C in a humidified 5% CO_2_ atmosphere. Transfection at 12 days *in vitro* (DIV) with a plasmid that carried EGFP under the control of a synapsin-1 promoter was performed with Lipofectamine reagent (Life Technologies). All of the experiments were performed at 18–21 DIV.

### Neuronal culture stimulation

Neuronal cultures were incubated for 40 min with either aaMMP-9 or iaMMP-9 in MM (400 ng/ml). The cells were then treated for 30 min with GM6001 (25 μM) dissolved in DMSO or treated with DMSO alone as a control. For cLTP in neuronal cultures, 50 μM forskolin, 50 μM picrotoxin, and 0.1 μM rolipram (Sigma-Aldrich; all dissolved in DMSO) were bath-applied (described previously). cLTP was applied to neurons in the presence or absence of iaMMP-9 (400 ng/ml) for 40 min. For the control experiments, hippocampal cultures were treated with DMSO.

### Live cell imaging

For imaging, 18–21 DIV hippocampal neurons were placed in an acquisition chamber under a controlled temperature and CO_2_ concentration. Segments of dendrites were imaged 10 min before stimulation. The neuronal cultures that were treated with aaMMP-9 or iaMMP-9 were imaged 40 min after protein application and then 30 min after inhibitor (GM6001) or solvent (DMSO) application. In control experiments the cells were incubated with the inhibitor (GM6001) and with the solvent of inhibitor (DMSO) for 70 min. The cLTP mixture with iaMMP-9 or DMSO was applied to the neuronal cultures by bath application. Segments of dendrites were imaged 10 and 40 min after stimulation. In treated cultures the final concentration of DMSO was 0.02% or lower. Such a low (0.02%) concentration of DMSO was shown not to affect the AMPA receptor motility, dendritic spine morphology and network activity^12^. All of the images were acquired using a confocal microscope with a PL Apo 40×/1.25 NA water immersion objective using a 488 nm diode-pumped solid-state laser at 10% transmission at a pixel count 1024 × 1024. A series of z-stacks were collected for each cell with a 0.42 μm step size. An additional digital zoom was applied, resulting in a lateral resolution of 0.07 μm per pixel.

### Analysis of dendritic spine shape

The confocal images were semiautomatically analyzed using SpineMagick software (patent no. WO/2013/021001). The dendritic spines that were analyzed belonged to secondary and ternary dendrites to reduce possible systematic differences in spine morphology that are caused by the location of spines on dendrites with different ranks. The recorded parameters were the spine length and head width. The spine length was determined by measuring the curvilinear length along a fitted virtual skeleton of the spine. The fitting procedure was performed by looking for a curve along which the integrated fluorescence was at a maximum. The head width was defined as the diameter of the largest spine section while excluding the bottom part of the spine (1/3 of the spine length adjacent to the dendrite). The spontaneous changes in dendritic spine shapes can obscure systematic effects. These changes can be explained by the spontaneous intrinsic fluctuation of dendritic spine shape^25^. In order to minimize this effect in the analysis of live-cell imaging, the same dendritic spines were identified in the images of different time points and the relative changes (i.e. (x1-x0)/x0) in length/width ratio were calculated and plotted using logarithmic scale. We used a scale-free parameter (the length/width ratio) and the head width, which reflect spine shape. The same dendritic spines were identified on images that were acquired during the live imaging sessions before and 10 and 40 min after stimulation and additionally 30 min after MMP inhibitor administration.

### Statistical analysis

The results are expressed as mean ± SEM. Groups of dendritic spines were compared using unpaired Student’s *t*-test or an unpaired *t*-test with Welch’s correction in cases in which the groups had significantly different variances. If the number of groups was larger than two, then a one-way ANOVA was performed.

## Additional Information

**How to cite this article**: Magnowska, M. *et al*. Transient ECM protease activity promotes synaptic plasticity. *Sci. Rep.* **6**, 27757; doi: 10.1038/srep27757 (2016).
